# Shifted LT Code Security Scheme for Partial Information Encryption

**DOI:** 10.3390/e24121776

**Published:** 2022-12-05

**Authors:** Si Zhang, Fanglin Niu, Lizheng Wang, Ling Yu

**Affiliations:** School of Electronics and Information Engineering, Liaoning University of Technology, Jinzhou 121001, China

**Keywords:** rateless code, partial information transfer, encryption, anti-eavesdropping, physical layer security

## Abstract

The existing physical layer security technology based on fountain codes needs to ensure that the legal channel is superior to the eavesdropping channel; when the quality of the legal channel and the eavesdropping channel are close, the information security cannot be guaranteed. Aiming at this problem, this paper proposes a shifted Luby transform (SLT) code security scheme for partial information encryption, which is mainly divided into two stages, partial information encryption transfer and degree distribution adjustment. The main idea is that the source randomly extracts part of the information symbols, and performs XOR encryption with the random sequence containing the main channel noise sent by the legitimate receiver. Afterward, the degree distribution is adjusted using the number of transfer information symbols received by the legitimate receiver to improve the average degree of the encoded codewords. Since the eavesdropper can only obtain fewer information symbols in the initial stage, it is difficult to decode the generated coded symbols after the degree distribution adjustment, thereby ensuring the safe transmission of information. The experimental results show that, compared with other LT anti-eavesdropping schemes, even if the legitimate channel is not dominant, the proposed scheme still has better security performance and less decoding overhead.

## 1. Introduction

Mobile communication is deeply affecting people’s work and life, and greatly promotes social and economic development. With the commercialization of 5G technology, the industry has also begun preliminary exploration of 6G, and the Internet of Everything will further flourish in 6G. However, the massive number of connected devices and the huge amount of transmitted data in 6G networks have brought great challenges to the realization of communication security. Compared with wireless transmission technologies such as Wi-Fi, Bluetooth, and ZigBee, 5G and 6G technologies have more complex protocol systems and control signaling, so their security measures are also more difficult.

Compared with the traditional upper-layer security technology Ref. [1], the security features provided by the physical layer have quantum-like characteristics such as lightweight, difficult to replicate, and high security. For example, by utilizing the randomness, time-variation, and uniqueness of wireless channels, channel state information can be quantified into a wireless key that is difficult to crack, and the security of the key does not depend on increasing the key length. At the same time, the differences between wireless channels can be exploited to achieve secure transmission of information through secure coding techniques Ref. [2]. At present, the main schemes to ensure the security of information transmission include: generating keys according to channel characteristics to encrypt information Refs. [3,4], multiuser cooperative work Ref. [5], artificial noise for signal processing Ref. [6], physical layer security coding Ref. [7], etc. Among them, the physical layer security coding scheme is of great research significance because it is simple to implement and can guarantee both security and reliability of information transmission. In the existing security coding method, as a rateless code, the fountain code has adaptability to the channel state, and has a small decoding overhead while ensuring the reliability of information transmission. Additionally, the fountain code has encoding randomness—the eavesdropper cannot obtain useful information directly from the leaked fountain code symbols, so for research the fountain code is widely used in the eavesdropping channel.

In 1998, Luby proposed the fountain code idea Ref. [8] and in 2002 gave a concrete and practical fountain code scheme called the Luby transform (LT) code Ref. [9]. It has the advantages of a simple encoding and decoding algorithm and low decoding overhead, and as a rateless code it can ensure successful decoding by the receiver even if the channel environment is poor. In order to further improve the performance of fountain code, scholars have conducted in-depth research. Ref. [10] used the information of the number of symbols recovered by the receiver to adjust the robust soliton distribution (RSD) degree distribution, and proposed a shifted LT (SLT) code, which significantly reduced the decoding overhead. Ref. [11] optimized the SRSD degree distribution function by reducing the rounding degree offset and the limiting probability distribution of degrees to further reduce the codec complexity. Ref. [12] combined analogue fountain codes (AFC) with density evolution (DE) and introduced differential evolution to optimize the parameters of the analogue fountain codes, for analyzing the performance of AFC decoder with infinite block lengths. In order to reduce the feedback overhead, in Ref. [13] the source estimates the receiver decoding situation and thus selects the appropriate degree for fountain coding. Ref. [14] found the basis of the fountain code using Gaussian elimination and identified the most reliable basis, which was used to recover the inactivated decoded source symbols. Ref. [15] introduced weighting factors to adjust the probability of occurrence of fountain-encoded symbols of different degrees in order to reduce the maximum buffer occupation and the number of feedback transmissions when decoded by the receiver.

Because fountain code is both rateless and coded randomly, which makes fountain codewords independent of one another and equally important; this is consistent with the starting point for secure coding using channel differences between legal and illegal channels, so that it is widely used in physical layer security transmission. Ref. [16] used a single-part graph to analyze the security performance of fountain codes, and proposed an optimal graph structure to achieve the minimum number of leaky symbols for online fountain codes, which was minimized by combining coset precoding. Ref. [17] dynamically adjusted the fountain coding structure through feedback channels and used transmit power control to improve the packet reception rate of legitimate receivers. Ref. [18] used collaborative jamming techniques to degrade the quality of the eavesdropping channel, wherein the collaborative jamming nodes take energy from the radio frequency signals of the source and the interferer to generate noise to the eavesdropper. In order to ensure data security, Ref. [19] introduced constellation rotation technology and interference noise into wireless signals to deteriorate the signal quality of eavesdroppers. Ref. [20] combined multicast and fountain codes to significantly reduce system latency and introduced collaborative relaying to improve system security. Ref. [21] used relay nodes to cooperatively jam the transmitted fountain codes and gave the secrecy probability and disruption probability of the system. Ref. [22] applied SLT in secure communication for research, and cascaded SLT code and LT code, which effectively improved the system security, but the proposed scheme had high computational complexity and higher requirements for the system’s encoding and decoding performance.

Although enhancing security by applying fountain codes has opened a promising area of research, most of the existing fountain code-based secure transmission schemes suffer from the following problems: First, multiple feedback of decoded information to achieve dynamic encoding not only increases system latency, but also risks the feedback being intercepted by eavesdroppers. Second, when collaborative diversity is used, it is difficult to guarantee the security of the system when the relay nodes are untrusted nodes. Cascade solutions, on the other hand, are computationally more complex and require higher hardware costs. All of the above schemes require physical layer security techniques to make the legitimate channel quality sufficiently advantageous, but security cannot be guaranteed when the legitimate channel is close to the quality of the eavesdropping channel.

Aiming at the above problems, this paper proposes a SLT code security scheme for partial information encryption and transfer. First, the legitimate receiver sends a random sequence used for encryption, and the source does not need to know the exact contents of the random sequence, but only needs to encrypt the received sequence containing noise with the transferred partial information to obtain the ciphertext, thus bringing the legitimate channel noise into the encrypted information sequence, so that the ciphertext intercepted by the eavesdropper is a degraded version of the legitimate receiver. Since the eavesdropper cannot obtain a complete random sequence and can only receive a small number of transferred ciphertext sequences, it is difficult for the eavesdropper to recover transferred partial information. Afterward, the source adjusts the degree distribution to improve the average degree of encoded symbols using the number of correctly transferred symbols already decrypted by the legitimate receiver. Since more transfer information is known to the legitimate receiver, the probability of the eavesdropper not completing the decoding is greatly increased when the legitimate receiver successfully decodes, which further increases the security of the system.

The main contributions of this article are as follows:

(1) In the scheme presented in this paper, the source randomly selects partial information symbols to send to the receiver as known information before coding and decoding. In order to prevent the eavesdropper from receiving part of the transferred information, the legitimate receiver sends a random sequence to the source for encryption. When the eavesdropper intercepts the information, legal channel noise is introduced. At the same time, in order to decrypt the information, the eavesdropper has to establish a channel with the legitimate receiver to steal random sequences, which further increases the eavesdropper’s uncertainty about confidential information.
(2) Secondly, the source adjusts the degree distribution using information on the number of transferred symbols decrypted by the legitimate receiver, reducing the probability of low-degree codewords that appear. Since the eavesdropper only intercepts a small number of transfer information symbols, the increase in the average degree of the codeword makes it difficult for the eavesdropper to decode.
(3) We evaluate the performance of the system by deriving the computational complexity of encoding and decoding along with the safe capacity, and the performance of the proposed scheme is analyzed and compared through simulation.

The rest of the paper is organized as follows. Section 2 introduces the related knowledge of SLT and degree distribution. Section 3 presents the SLT code secure communication scheme based on partial information encryption transfer. Section 4 provides a theoretical analysis of the encoding and decoding performance and system security of the proposed scheme. Section 5 presents the simulation results. Finally, we conclude this paper in Section 6.

## 2. SLT Code and Degree Distribution

### 2.1. SLT Code Encoding and Decoding Process

As a channel coding technology, the SLT code Ref. [10] first selects some original symbols and sends them directly to the receiver as known information without coding. The source uses the information about the number of symbols known to the receiver to adjust the RSD degree distribution to obtain the SRSD degree distribution function. According to the SRSD degree distribution function, d symbols are randomly selected from the source symbols for XOR operation to obtain the corresponding SLT coded symbols, and the belief propagation (BP) algorithm is used for decoding.

The SLT code encoding and decoding process is as follows:

(a) Encoding process
  (1) First, the source randomly selects m(0≤m≤k) partial information symbols from the k original symbols and sends them directly without encoding. The legitimate receiver feeds back the number n(0≤n≤m) of correct symbols received to the source.
  (2) The source uses n to adjust the RSD degree distribution to obtain the SRSD degree distribution. A degree d is taken out from the degree distribution function, and then d of the k original symbols are randomly selected for the XOR operation, and the result is the SLT codeword in that time slot.
  (3) Repeat the above process to generate a continuous stream of SLT codewords.
(b) Decoding process
  The receiver XORs the received SLT codes with the known information symbols and disconnects them from the original symbols. Repeat this process until the remaining k−n unknown symbols are recovered.

When the traditional fountain code performs BP decoding, it must receive the coded symbol of degree 1 to start decoding. For the SLT code with partial information transfer, the receiver knows part of the information, that is, there are a certain number of original information symbols of degree 1, so the decoding time is earlier than that of the traditional fountain code, and the decoding speed is faster.

### 2.2. Degree Distribution of SLT Codes

The SRSD degree distribution used for SLT codes is obtained by shift adjustment of the RSD degree distribution using the number n of partial symbols known to the receiver Ref. [10]. The SRSD degree distribution makes the probability of occurrence of low degrees in RSD decrease, increases the probability of occurrence of other degrees, expands the coverage of the original symbols, and significantly reduces the number of codewords required by the receiver to decode. The SRSD degree distribution is shown in Formula (1):

(1) $$\gamma (j)=\mu_{RSD(k-n)}(d),\;\;j=round\left(\frac{d}{1-n/k}\right),\;1\leq j\leq k$$

where k is the number of original symbols in each group, n is the number of correct information symbols known to the legitimate receiver, d represents the degree of the coded symbol before the degree distribution adjustment, j represents the new degree obtained after the degree distribution adjustment, round(*) denotes rounding, and $\mu_{RSD}$ denotes the RSD degree distribution.

The RSD degree distribution function is:

(2) $$\begin{array}{ll} \mu \left(d\right)=\frac{\rho \left(d\right)+\tau \left(d\right)}{z}, & d=1,2,\cdots ,k \end{array}$$

where $z=\sum_{d}\left(\rho \left(d\right)+\tau \left(d\right)\right)$, ρ(d) is the ideal soliton distribution function, τ(d) is the enhancement factor.

(3) $$\rho (d)=\{\begin{array}{ll} 1/k & d=1\\ \frac{1}{d(d-1)} & d=2,3,\cdots ,k \end{array}$$

(4) $$\tau (d)=\{\begin{array}{ll} s/\left(k\cdot d\right) & d=1,2,3,\cdots ,\left(k/s\right)-1\\ \frac{s}{k}\ln(s/\delta ) & d=k/s\\ 0 & d>k/s \end{array}$$

where $s=c\ln\left(k/\delta \right)\sqrt{k}$, c is a constant, σ represents the maximum allowed probability of decoding failure.

By normalizing the SRSD degree distribution of Formula (1), a normalized SRSD degree distribution function based on partial information can be obtained, as shown in Formula (5):

(5) $$R_{SRSD}\left(j\right)=\frac{\gamma_{\left(k-n\right)}(j)}{\sum_{j}\gamma_{\left(k-n\right)}(j)}$$

If the source knows the number n of information symbols recovered by the receiver, the fountain code obtained by bringing n into the SRSD degree distribution function of Formula (5) is called the SLT code.

### 2.3. SRSD Degree Distribution Rounding Offset

In the SLT code, the receiver has received n transferred information symbols, that is, a certain number of degree 1 codewords already exist, so the source does not need to send too many degree 1 coded symbols for decoding. Therefore, in the SRSD degree distribution obtained by the RSD shift, the probability distribution of degree 1 is reduced or equal to 0. While reducing the probability distribution of low degrees, the probability of occurrence of other degrees is increased, the correlation between coded symbols is increased, and the decoding overhead of fountain codes is reduced. However, if we let each coded cover all source symbols, then the coded symbols will reach the largest correlation. However, there is no way to decode anything.

**Figure 1:**
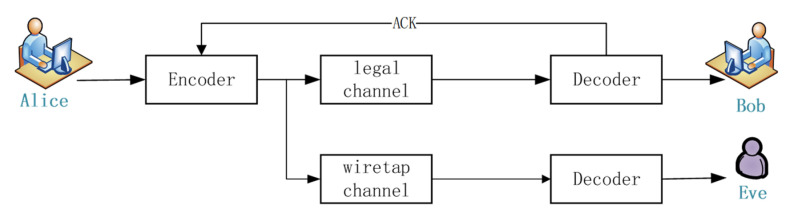
Wyner wiretap channel model.

Ref. [11] pointed out that since the degree value represents the number of symbols selected, there is a rounding operation in the adjustment process of the SRSD degree distribution, and the actual degree j and the ideal degree $j_{ideal}$ obtained by rounding will produce an error offset. It is pointed out that when n=0.2×k, the accumulated error offset of $R_{SRSD}(j)$ reaches the maximum, which leads to an increase in the number of decoded symbols. Ref. [22] applied the SLT code to the Wyner channel model for research, and found that the value of n would affect the system decoding overhead and eavesdropper error rate. The bit error rate of the eavesdropper shows a sawtooth change with the increase in n, and the peak value of the sawtooth gradually decreases. The Wyner channel model is shown in Figure 1.

In Figure 1, Alice is the legitimate sender, Bob is the legitimate receiver, and Eve is the eavesdropper. Ref. [22] pointed out that when SLT is used as the secure coding method, the bit error rate of the eavesdropper is higher when the legitimate receiver receives n=0 and 0.2k transferred information symbols. When n=0, the SLT code degenerates into the traditional LT code. When n= 0.2k, the rounding error offset of the degree distribution reaches the maximum, which increases the average degree. The eavesdropper needs more codewords to complete the decoding, and the system security increases. In general, when *n* = 0.2*k*, it can not only effectively reduce the decoding cost of the system, but also ensures the safe transmission of information.

Therefore, the premise of the scheme presented in this paper is to ensure that the legitimate receiver can receive n= 0.2k transferred information symbols as known information. Owing to the influence of channel quality, in a legal channel with erasure probability $P_{AB}$, in order to ensure that n correct random symbols can be received, Alice needs to randomly select $m=n/\left(1-P_{AB}\right)$ information symbols from k original symbols for transfer.

## 3. Design of SLT Code Security Scheme for Partial Information Encryption and Transfer

According to the above analysis, the SLT code is mainly divided into two parts, one is to randomly select some of the original information symbols for transfer, and the other is to adjust the degree distribution according to the number of correctly transferred information symbols received by the legitimate receiver. It is easy to see that the transfer of partial information has a significant impact on the performance of SLT. If the eavesdropper can only obtain a small amount or cannot acquire transferred information symbols at all, then due to the subsequent adjustment of the degree distribution makes the average degree of the codewords increase, it is difficult for the eavesdropper to obtain degree 1 codewords for decoding, the contribution rate to the subsequent decoding is significantly reduced, and the security of the system is better guaranteed.

Based on this, this paper considers sending a random sequence to the source at the legitimate receiver Bob, and the source uses the received random sequence with legitimate channel noise to XOR the m information symbols to be transferred in the SLT to obtain the ciphertext. Alice does not need to know the specific content of the random sequence. Since Bob knows the random sequence, Bob can directly decrypt the received ciphertext sequence. The eavesdropper Eve introduces legal channel noise while stealing the encrypted transfer information. Owing to the influence of eavesdropping channel noise and legal channel noise, the uncertainty of transfer information increases. At the same time, it is difficult for Eve to obtain the correct random sequence, so it is difficult to decrypt the intercepted ciphertext information and the contribution to subsequent decoding decreases rapidly. Moreover, the adjustment of the degree distribution of the SLT code reduces the probability of low degree occurrence, and it is more difficult for Eve to decode the subsequent codewords. The security performance of wireless communication is further improved.

### 3.1. Encryption of Transfer Information

**Figure 2:**
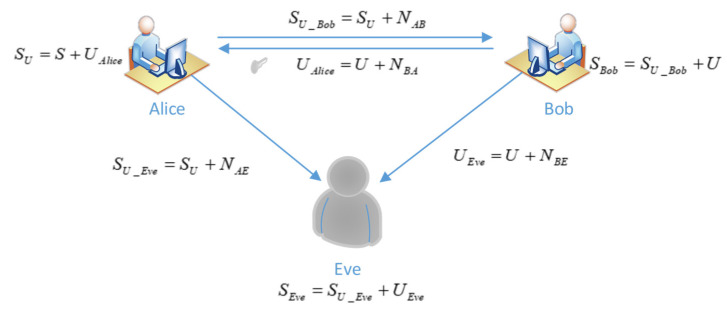
Partial information encrypted transfer.

The model described in this paper is shown in Figure 2. Alice is the legitimate sender of information, Bob is the legitimate receiver, and Eve is the eavesdropper. The channel between Alice and Bob is a legal channel, the forward transmission noise from Alice to Bob is $N_{AB}$, and the reverse transmission noise from Bob to Alice is $N_{BA}$. Before the communication starts, Alice needs to send a pilot signal to tell Bob to prepare for communication. When Bob receives the pilot signal, he can estimate the legal channel noise $N_{AB}$ at this time, and at the same time sends a group of random sequences U used for partial information symbol encryption to Alice. The eavesdropper Eve can only conduct passive eavesdropping; The channel between Alice and Eve is called the eavesdropping channel and its channel noise is denoted as $N_{AE}$. In order to perform decoding, Eve also needs to receive the random sequence U sent by Bob, thereby establishing an eavesdropping channel between Bob and Eve, and introducing the noise $N_{BE}$ between them into Eve at the same time. The specific model is shown in Figure 2.

During information transmission, because of the influence of channel noise, some information symbols will be wrong, and will be erased when the receiver cannot judge whether the information symbols are correct. Denote the legal channel erasure probability as $P_{AB}$, the forward transmission noise as $N_{AB}$, and the reverse transmission noise as $N_{BA}$. Bob estimates $N_{AB}$ through the pilot signal contained in Alice’s transmitted signal. For the scheme described in this paper, Eve can only passively eavesdrop, the erasure probability of eavesdropping channel is $P_{AE}$, $P_{BE}$, respectively, and the noise is $N_{AE}$, $N_{BE}$, respectively, the communication channel is a binary erasure channel (BEC), subject to slow fading characteristics. The source symbols are grouped, and each group has *k* symbols $X=\left(x_{1},\;x_{2},\;\cdots \;,\;x_{k}\right)$. 

The scheme in this paper focuses on confidentiality when performing partial information transfer, and Bob needs to send a random sequence for partial information encryption first. This process is discussed and analyzed in detail below.

(1) When Bob receives the communication request signal, a series of random binary sequences $U=\left(u_{1},\;u_{2},\;\cdots ,\;u_{m}\right)$ are generated immediately. The length of the sequence U is the same as the length of the partial information sequence $S=\left(s_{1},\;s_{2},\;\cdots ,\;s_{m}\right)$ selected by the source, and the U broadcast is sent to Alice.
(2) Alice and Eve receive a random sequence with noise broadcasted by Bob at the same time. Since the legitimate channel and the eavesdropping channel are different channels, even if the erasure probabilities of both are the same, the noisy random sequences received by both sides are different. where the random sequence received by Alice is:
  (6) $$U_{Alice}=U+N_{BA}$$
  where $U_{Alice}$ represents the random sequence containing legitimate channel noise received by Alice, and $N_{BA}$ is the channel noise from Bob to Alice.

The random sequence received by Eve is:

(7) $$U_{Eve}=U+N_{BE}$$

where $U_{Eve}$ represents the random sequence containing the channel noise of the corresponding channel received by Eve, and $N_{BE}$ is the channel noise from Bob to Eve.

Formulas (6) and (7) are both the superposition of binary signals and noise signals. When the receiver cannot judge whether the received symbol is 0 or 1, it will be erased, and the position i of the erased symbol will be recorded. The erase symbol is set to $N_{error\_i}$.

Alice randomly selects m information symbols $S=\left(s_{1},\;s_{2},\;\cdots ,\;s_{m}\right)$ from the k original symbols for transfer. Under the BEC channel, in order to ensure that Bob receives $n_{Bob}=0.2k$ correct information symbols as known information, the length m of the selected sequence S should not be less than $0.2k/\left(1-P_{AB}\right)$. Alice performs XOR operation on the information sequence S and the random sequence $U_{Alice}$. When encountering the previous erasure symbol $N_{error\_i}$, in order to ensure that the noise signal can be merged into the source symbol, the following conditions need to be satisfied:

(8) $$S\left(i\right)+{\left(N_{BA}\right)}_{error\_i}={\left(N_{BA}\right)}_{error\_i}$$

Then, the ciphertext sequence $S_{U}$ is obtained, and $S_{U}$ is sent to Bob by broadcasting. The transmitted signal at this time is expressed as:

(9) $$\begin{array}{ll} S_{U} & =S+U_{Alice}\\ & =S+U+N_{BA} \end{array}$$

(3) Bob and Eve will receive the ciphertext sequence sent by Alice. Owing to the influence of channel noise, the signals received by Bob and Eve are, respectively:
  (10) $$\begin{array}{ll} S_{U\_Bob} & =S_{U}+N_{AB}\\ & =\left(S+U+N_{BA}\right)+N_{AB} \end{array}$$
  (11) $$\begin{array}{ll} S_{U\_Eve} & =S_{U}+N_{AE}\\ & =\left(S+U+N_{BA}\right)+N_{AE} \end{array}$$
(4) When Bob receives $n_{Bob}=0.2k$ correct information symbols, the number $n_{Bob}$ is fed back to the source and Alice stops sending partial information. Bob and Eve decrypt the received ciphertext sequence. From Formulas (10) and (11), it can be known that the broadcast signals received by Bob and Eve are $S_{U\_Bob}$ and $S_{U\_Eve}$, respectively. Since Bob knows the random sequence U, which can be directly brought into recovery, Eve can only rely on $U_{Eve}$ for decryption. Specifically, this is described as:
  (12) $$\begin{array}{ll} S_{Bob} & =S_{U\_Bob}+U\\ & =\left(S+U+N_{BA}\right)+N_{AB}+U \end{array}$$
  (13) $$\begin{array}{ll} S_{Eve} & =S_{U\_Eve}+U_{Eve}\\ & =\left(S+U+N_{BA}\right)+N_{AE}+U+N_{BE} \end{array}$$

Since Bob knows $N_{AB}$, it can be concluded from Formulas (13) and (14) that the main noise contained in Bob’s signal is:

(14) $$N_{Bob}=N_{BA}$$

The noise contained in the eavesdropper Eve’s signal is:

(15) $$N_{Eve}=N_{AE}+N_{BA}+N_{BE}$$

It can be known from Formulas (14) and (15) that $N_{AE}+N_{BE}+N_{BA}\geq N_{AB}$ exists regardless of the quality of the legitimate channel and the eavesdropping channel. At this time, on the premise of the same source information, the noise at Eve’s end in the received signal is greater than that at Bob’s end. The information received by Eve is a degraded version of Bob, and the interception probability of Eve decreases, decreasing the contribution to the subsequent decoding.

### 3.2. Scheme Process

Assuming that Bob and Eve have the same decoding rules, the communication channel is a binary erasure channel (BEC), subject to slow fading characteristics. The erasure probabilities of legitimate channel and eavesdropping channel are $P_{AB}$ and $P_{AE}$, respectively. The source symbols are grouped, and each group has k symbols $X=\left(x_{1},\;x_{2},\;\cdots \;,\;x_{k}\right)$. The specific steps based on the SLT code encryption scheme are as follows:

(1) Alice sends a pilot signal to notify Bob to prepare for communication, and then Bob sends a random binary sequence $U=\left(u_{1},\;u_{2},\;\cdots ,\;u_{m}\right)$ of length m to Alice.
(2) Alice randomly selects m symbols $S=\left(s_{1},\;s_{2},\;\cdots ,\;s_{m}\right)$ (S⊂X) from the k original symbols and performs XOR operation with the received random sequence $U_{Alice}$ to obtain the ciphertext sequence $S_{U}$, and sends $S_{U}$ to Bob by broadcasting.
(3) Bob decrypts the received ciphertext $S_{U\_Bob}$ and the key sequence U by XOR operation. When Bob recovers $n_{Bob}=0.2k$ correct information symbols, he feeds back the number $n_{Bob}$ to the source, and tells the source to stop sending the ciphertext sequence.
(4) Alice uses $n_{Bob}$ to adjust the RSD degree distribution to obtain the SRSD degree distribution, which is based on the encoding process of SLT to obtain the codeword C and send it to Bob.
(5) Bob uses the known information symbols to perform BP decoding on the received SLT codeword $C_{Bob}$, and sends ACK when all the k original symbols are recovered to inform Alice to stop sending this group of codewords.
(6) Alice receives the ACK, stops sending this group of codewords, and prepares to send the next group of source symbols.
(7) Repeat steps (1) to (6) until the source symbols of all groups are recovered.

To summarize, in the proposed scheme, during the partial information transfer stage, Bob sends a random sequence via broadcast, and the difference between the eavesdropping channel and the legitimate channel makes the noisy random sequences received by Alice and Eve different. Alice uses Alice’s own $U_{Alice}$ to encrypt the transferred partial information, while Eve can only decrypt it by the received $U_{Eve}$. This encryption method enables the sender to superimpose the legitimate channel noise on Eve, and the difference between $U_{Alice}$ and $U_{Eve}$ of the noisy random sequence enables Bob to obtain more valid information symbols than Eve, achieving the effect of anti-eavesdropping.

In the encoding and decoding stage, since it is difficult for Eve to recover the encrypted transition information symbols and the degree distribution is adjusted according to the transition information received by Bob, the average degree of codewords increases. At the same time, according to the random packet loss characteristics of fountain code under BEC channel, with the continuous encoding and decoding, the information gap between Bob and Eve is increasing, and the probability of Eve decoding success is rapidly decreasing. When Bob has recovered all the messages, Bob sends an ACK command to inform Alice not to send any more coded symbols, so that Eve cannot intercept more codewords for decoding, thus effectively ensuring the security of wireless communication.

For the scheme described in this paper, firstly, it is necessary to encrypt and transfer some of the randomly selected original information symbols, and then adjust the degree distribution according to the number of correct transfer information symbols received by Bob. Since the degree distribution adjustment process is introduced in detail in Section 2.2, we focus below on the specific implementation method of partial information encryption transfer.

## 4. Theoretical Analysis

In this section, we theoretically analyze the encoding and decoding performance of the proposed scheme and the security performance of the system, and verify the performance of the proposed scheme through theoretical derivation.

### 4.1. Computational Complexity of Encoding and Decoding

The proposed scheme is divided into two stages: partial information transfer stage and encoding and decoding stage. Assuming that the erasure probability of the legal channel is $P_{AB}$, the erasure probability of the eavesdropping channel is $P_{AE}$ and $P_{BE}$, respectively, and the number of transmitted symbols of each source is k. Under the BEC channel, when partial information is transferred, for Bob, the information transmission is affected by the legal channel. For Eve, the amount of intercepted information is related to the eavesdropping channel and the legal channel environment. Next, we analyze the complexity of the system through the number of XOR operations required for encoding and decoding.

In the encoding and decoding stages, due to partial information transfer and Bob’s known $n_{Bob}$ information symbols, to recover the remaining $k-n_{Bob}$ original symbols, at least $k-n_{Bob}$ correctly encoded symbols need to be received. Owing to the random packet loss characteristics of the erasure channel, the source must send at least $\left(k-n_{Bob}\right)/\left(1-P_{AB}\right)$ SLT codewords so that Bob can receive $k-n_{Bob}$ correctly coded symbols. According to Ref. [10], for SLT codes, the average degree of each coded symbol ${\bar{d}}_{SLT}=O\left(\frac{k}{k-n_{Bob}}\ln(k-n_{Bob})\right)$. Therefore, we can find that, for a fixed decoding failure probability δ and when using the proposed scheme for encoding, Bob needs at least the number of XOR operations $L_{E\_Bob}$ to satisfy:

(16) $$\begin{array}{l} L_{E\_Bob}=\frac{k-n_{Bob}}{1-P_{AB}}{\bar{d}}_{SLT}\\ \;\;\;\;=\frac{k-n_{Bob}}{1-P_{AB}}\cdot \frac{k}{k-n_{Bob}}\ln\left(k-n_{Bob}\right)\\ \;\;\;\;=\frac{k}{1-P_{AB}}\ln\left(k-n_{Bob}\right) \end{array}$$

where ${\bar{d}}_{SLT}$ denotes the average degree of the SLT code.

Further, the number $L_{D\_Bob}$ of XOR operations that Bob needs to perform when decoding is expressed as

(17) $$L_{D\_Eve}=\left(k-n_{Eve}\right){\bar{d}}_{SLT}$$

From Formulas (16) and (17), it can be seen that the computational complexity of Bob and Eve is related to the number of partial messages $n_{Bob}$, $n_{Eve}$ that they receive in the initial stage. In the partial information transfer stage, the number of information $n_{Bob}$ and $n_{Eve}$ that Bob and Eve can receive satisfies:

(18) $$n_{Bob}=m\times \left(1-P_{AB}\right)$$

(19) $$n_{Eve}=m\times \left(1-P_{AB}\right)\left(1-P_{AE}\right)\left(1-P_{BE}\right)$$

It can be known from this formula that for the scheme described in this paper, $n_{Bob}\geq n_{Eve}$ is always established, therefore, $L_{D\_Bob}\leq L_{D\_Eve}$ can be obtained, the number of operations required by the eavesdropper to complete the decoding is more, and the computational complexity is greater.

In practice, since Eve can only passively steal information, the number of codewords that Eve can intercept is related to Bob. When Bob completes decoding, the actual number of codewords $m_{SLT\_Eve}$ that Eve can receive satisfies:

(20) $$m_{SLT\_Eve}=\frac{1-P_{AE}}{1-P_{AB}}\left(k-n_{Bob}\right)$$

Let $S_{r}=\left(1-P_{AE}\right)/\left(1-P_{AB}\right)$, compared with the ideal case when the number of codewords needed to complete decoding is $m_{ideal\_Eve}=k-n_{Eve}$, it is not difficult to see:

(21) $$\left\{ \begin{array}{l} m_{ideal\_Eve}>m_{SLT\_Eve},\;0\leq S_{r}\leq 1\\ m_{ideal\_Eve}\geq m_{SLT\_Eve},\;1<S_{r}\leq \left(k-n_{Eve}\right)/\left(k-n_{Bob}\right)\\ m_{ideal\_Eve}<m_{SLT\_Eve},\;\left(k-n_{Eve}\right)/\left(k-n_{Bob}\right)<S_{r} \end{array}\right.$$

When $0\leq S_{r}\leq 1$, the eavesdropping channel is worse than the legal channel, and the actual number of codewords that Eve can receive is less than the number of codewords required for ideal decoding, that is, $m_{SLT\_Eve}<m_{ideal\_Eve}$; Eve cannot receive enough codewords to recover the original symbol. When $1<S_{r}\leq \left(k-n_{Eve}\right)/\left(k-n_{Bob}\right)$, the eavesdropping channel is better than the legal channel and Eve can intercept more codewords, but due to the partial information symbols received by Eve in the initial stage $n_{Eve}<n_{Bob}$, therefore, when Bob’s decoding is complete, Eve still has difficulty recovering the original information. When $\left(k-n_{Eve}\right)/\left(k-n_{Bob}\right)<S_{r}$, the quality of the eavesdropping channel is better. Compared with the previous two cases, although $n_{Eve}$ is still smaller than $n_{Bob}$, $n_{Eve}$ has increased, and the codewords actually intercepted by Eve are more than the ideal situation, that is, $m_{SLT\_Eve}>m_{ideal\_Eve}$, Eve can complete decoding at the same time or even before Bob. It is not difficult to see that the scheme proposed in this paper can not only ensure the security of information when the legal channel is better, but even if the quality of the legitimate channel and the eavesdropping channel is the same or the eavesdropping channel is even better, then the scheme in this paper can still ensure that the legitimate receiver completes the decoding first. When the eavesdropper intercepts less partial information at the initial stage, the security performance of the scheme described in this paper is better, and the security of information is still better guaranteed when the quality of the legitimate channel is close to that of the eavesdropping channel.

### 4.2. System Security

To measure the security performance of the system, we discuss the security capacity (CS) of the proposed scheme. The security capacity is described as the maximum information transmission rate while guaranteeing the security transmission, that is, $C_{S}=\max R=\max\left(R_{Bob}-R_{Eve}\right)$. For the scheme described in this paper, the information transmission rate needs to be analyzed in three stages, namely, random sequence transmission, partial information encryption transfer and SLT code transmission. In the random sequence transmission stage, Bob knows the complete random sequence U, and Eve steals the sequence $U_{Eve}$ through the eavesdropping channel $P_{BE}$. According to Shannon’s information theory Ref. [1], we can obtain that under the BEC channel, the random sequence information rates $R_{U\_Bob}$ and $R_{U\_Eve}$ at the Bob and Eve ends satisfy, respectively:

(22) $$R_{U\_Bob}=H\left(U\right)$$

(23) $$R_{U\_Eve}=I\left(U;\;U_{Eve}\right)=1-P_{BE}$$

Alice generates the ciphertext $S_{U}$ by XORing the partial information sequence S with the received random sequence $U_{Alice}$ containing noise, and sends it to Bob, and Eve steals it through the eavesdropping channel $P_{AE}$. Bob and Eve use their respectively known random sequences for decryption, and the transmission rates $R_{S\_Bob}$ and $R_{S\_Eve}$ of their partial information satisfy, respectively:

(24) $$\begin{array}{l} R_{S\_Bob}=I\left(S_{U};\;S_{U\_Bob}\right)\times R_{U\_Bob}\\ \;\;\;\;\;=I\left(S_{U};\;S_{U\_Bob}\right)\times H\left(U\right)\\ \;\;\;\;\;\leq \left(1-P_{AB}\right)\times \log_{2}\left(m\right) \end{array}$$

(25) $$\begin{array}{l} R_{S\_Eve}=I\left(S_{U};\;S_{U\_EVE}\right)\times R_{U\_Eve}\\ \;\;\;\;\;=\left(1-P_{AE}\right)\left(1-P_{AB}\right)\times \left(1-P_{BE}\right) \end{array}$$

It is easy to see that because $R_{U\_Bob}>R_{U\_Eve}$, and $I\left(S_{U};\;S_{U\_Bob}\right)>I\left(S_{U};\;S_{U\_EVE}\right)$, so $R_{S\_Bob}\gg R_{S\_Eve}$, it is difficult for Eve to recover the transferred partial information symbols. When Bob receives $n_{Bob}=0.2k$ partial information symbols, the source uses $n_{Bob}$ to adjust the RSD degree distribution, and then obtains the SLT codeword C and transmits it. Bob and Eve decode the received codewords $C_{Bob}$ and $C_{Eve}$ using their respectively known partial information symbols. The information transmission rates $R_{Bob}$ and $R_{Eve}$ of the SLT codeword by Bob and Eve satisfy, respectively:

(26) $$\begin{array}{l} R_{Bob}=I\left(C;\;C_{Bob}\right)\times R_{S\_Bob}\\ \;\;\;\;=\left(1-P_{AB}\right)\times R_{S\_Bob} \end{array}$$

(27) $$\begin{array}{l} R_{Eve}=I\left(C;\;C_{Eve}\right)\times R_{S\_Eve}\\ \;\;\;\;=\left(1-P_{AE}\right)\times R_{S\_Eve} \end{array}$$

When Bob completes decoding, Bob sends feedback to inform the source Alice to stop sending this group of codewords, defining the coefficient $S_{r}=\left(1-P_{AE}\right)/\left(1-P_{AB}\right)$, at which time the system security rate R satisfies:

(28) $$\begin{array}{l} R=R_{Bob}-R_{Eve}\\ \;\;\;=\left(1-P_{AB}\right)\times R_{S\_Bob}-\left(1-P_{AE}\right)\times R_{S\_Eve}\\ \;\;\;=\left(1-P_{AB}\right)\left(R_{S\_Bob}-S_{r}\times R_{S\_Eve}\right) \end{array}$$

Because $0\leq \left(1-P_{AB}\right)\leq 1$, in order to guarantee R>0, $S_{r}$ needs to satisfy $S_{r}<\left(R_{S\_Bob}/R_{S\_Eve}\right)$. Additionally, because $R_{S\_Bob}\gg R_{S\_Eve}$, therefore $\left(R_{S\_Bob}/R_{S\_Eve}\right)>1$. It can be seen that as $S_{r}$ increases, R gradually decreases. In particular, when $S_{r}\to 0$, $P_{AE}\to 1$, the quality of the eavesdropping channel is poor, it is difficult for Eve to steal the transmitted confidential information, and the confidentiality rate R reaches the maximum, which is close to the Shannon capacity limit. When $0<S_{r}<1$, $P_{AB}<P_{AE}$, the legal channel environment is better than the eavesdropping channel, and the security is very good at this time. When $S_{r}=1$, the quality of the legitimate channel is the same as that of the eavesdropping channel, but the secure transmission of information can still be guaranteed. In particular, when $1<S_{r}<\left(R_{S\_Bob}/R_{S\_Eve}\right)$, the quality of the eavesdropping channel is better than that of the legitimate channel, but it is difficult for Eve to recover the encrypted information symbols when partial information is transferred, so the security of the system can still be guaranteed.

## 5. Simulation and Analysis

In this section, the performance indexes of the proposed scheme are simulated and verified. From the theoretical analysis in Section 4, it can be clearly seen that the length of the transmitted symbol sequence, the channel erasure probability, and the change in the degree distribution function affect the performance of the system. Therefore, we observe the influence of the changes in various parameters on the system performance through simulation. When Alice sends 2000 groups of symbols, the number of information symbols in each group is k=300 and the parameters c=0.03 and δ=0.5 in the RSD and SRSD degree distributions. We compare this with the LT scheme, the SLT scheme, and the SLT-LT cascade scheme in Ref. [22]. Considering that there is only one eavesdropping channel in each stage of information transmission, for the convenience of analysis and discussion, we assume that $P_{AE}=P_{BE}$, and Eve and Bob have the same decoding rules, and use Matlab for simulation.

### 5.1. Information Recovery Process

**Figure 3:**
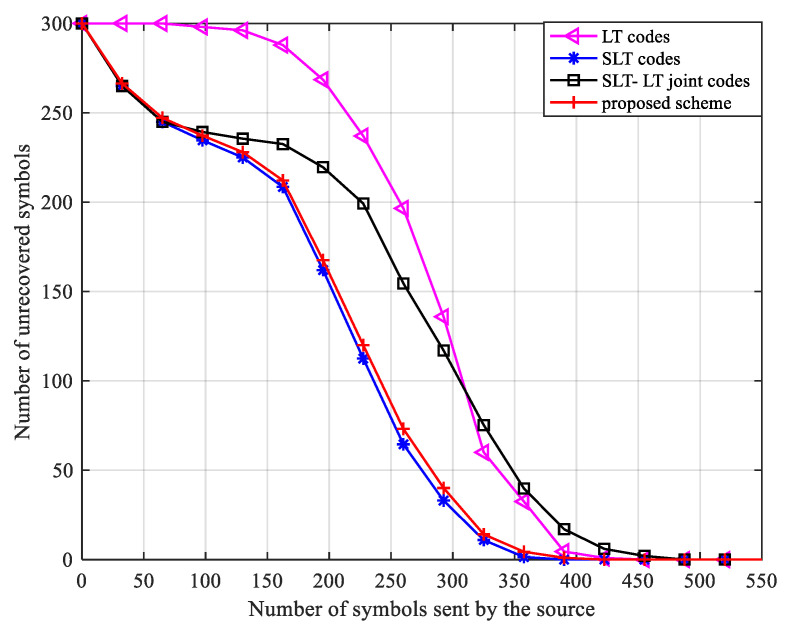
The relationship between the number of unknown symbols of the receiver and the number of symbols sent by the source.

Let the number of information symbol in each group k=300, the legal channel erasure probability $P_{AB}=0.3$, in the SLT, SLT-LT schemes, and the scheme in this paper, $n_{Bob}=60$. Figure 3 shows the relationship between the number of symbols sent by the source and the number of unrecovered input symbols by the receiver.

It can be seen from the change curve in Figure 3 that when the receiver receives fewer symbols, the number of input symbols that can be recovered is extremely small. When the number of received symbols reaches a certain range, the number of symbols decoded by the receiver begins to increase rapidly. Because conventional LT codes do not perform partial information transfer, decoding can be started only when the codeword with degree 1 is received. Owing to the transfer of part of the information, between the scheme in this paper and the SLT and SLT-LT schemes, the receiver received 0.2k degree 1 symbols, so the decoding time starts from 0. However, in this scheme, since the encryption and decryption process has a certain impact on the transferred part of the information, in the partial information transfer stage, compared with the SLT scheme, this scheme needs to receive slightly more information symbols to receive 0.2k symbols, which makes the overall decoding process slightly backward. However, in the SLT-LT concatenated coding scheme of the Ref. [22], since concatenated coding and decoding are required, the coding and decoding complexity is relatively high, and more codewords are required to complete the decoding.

### 5.2. Decoding Overhead

**Figure 4:**
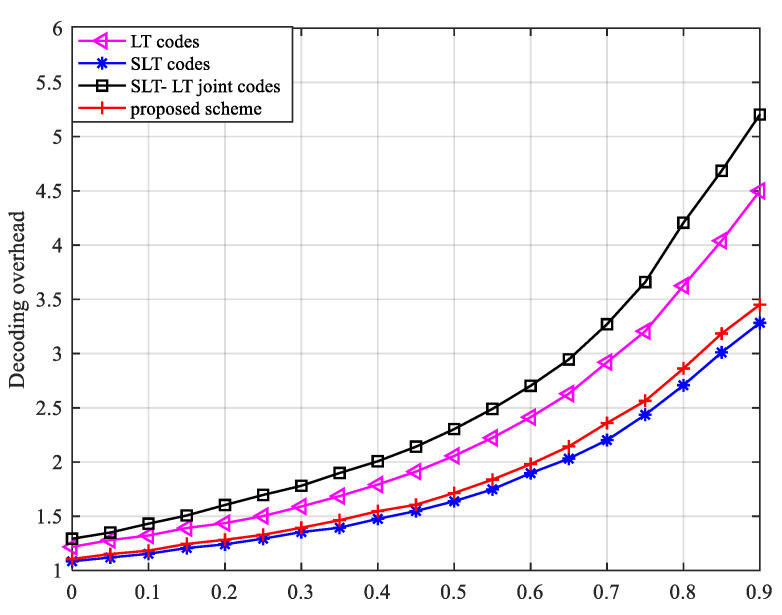
The relationship between the decoding overhead of legal receiver Bob and the erasure probability of the main channel.

The simulation parameters remain unchanged, ensuring that Bob receives $n_{Bob}=60$ correct symbols, $P_{AB}\in [0,\;0.9]$. Figure 4 gives the decoding overhead curve for legitimate receivers when the probability of legitimate channel erasure varies.

As can be seen from Figure 4, because of the transfer of some information and the encryption process, the number of coding symbols required by the proposed scheme is smaller than that of the traditional LT code and slightly larger than that of the SLT code. Compared with the SLT-LT concatenated coding scheme, the decoding overhead of the proposed scheme is greatly reduced. When the probability of channel erasure is small, fewer codewords are erased, so the receiver can receive more correct codewords in the same time, and the decoding overhead of the four schemes is small. As the probability of channel erasure increases, the channel environment gradually deteriorates, and the probability of the transmitted codewords being erased increases, so that all four schemes need to receive more codewords to recover the original information. However, the SLT-LT scheme has the largest decoding overhead due to the need for concatenated coding. With the increase in the erasure probability, the SLT-LT scheme has the fastest increase in the decoding overhead.

### 5.3. Eavesdropper BER

The scheme described in this paper combines the upper layer encryption technology to bring the legitimate channel interference into the eavesdropping channel, aiming to use the existing security technology to reduce the number of input symbols recovered by the eavesdropper as much as possible to achieve secure transmission. Considering from the Eve side, in the mechanism of this paper, it is the quality difference between the eavesdropping channel and the legitimate channel that affects the input symbol decoding rate at the Eve side; therefore, we observe the relationship between the channel environment and the BER of the eavesdropper Eve through simulation.

**Figure 5:**
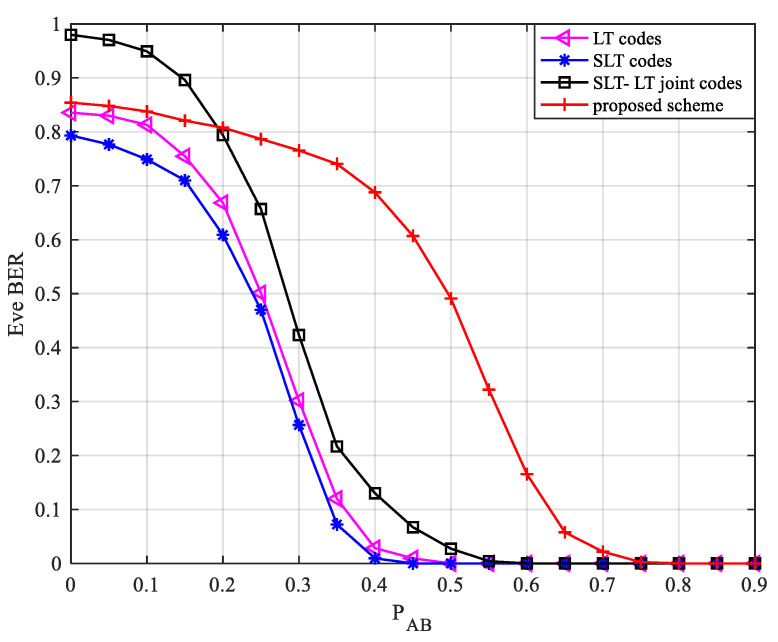
Relationship between Eve BER and erasure probability of the legal channel.

Let $P_{Eve}=0.3$, $P_{AB}\in [0,\;0.9]$; Figure 5 shows the change in the bit error rate of the eavesdropper under the change in the legal channel erasure probability F of the traditional LT scheme, the SLT scheme, the SLT-LT cascade scheme, and the scheme in this paper.

It can be seen from Figure 5 that the recovery probability of the eavesdropper under the scheme described in this paper is greatly reduced. When $P_{AE}$ remains unchanged, with the increase in $P_{AB}$, the Eve BER of the four schemes decreases. When $P_{AB}\leq P_{AE}$, the BER of the eavesdropper Eve is larger, and the four schemes can achieve better security performance.

When $P_{AB}>P_{AE}$, the legal channel is worse than the eavesdropping channel. Since the information symbols obtained by Eve in the initial stage of the scheme described in this paper are less, when the quality of the legal channel is close to the eavesdropping channel or the quality of the eavesdropping channel is even better, then Eve still has a large bit error rate. With the increase in the legitimate channel erasure probability, Eve can intercept more codewords under the four schemes, and the BER decreases. As $P_{AB}$ increases, the BER gradually approaches 0. Comparing the four schemes, when the legal channel is not dominant, the scheme proposed in this paper still has good security performance.

**Figure 6:**
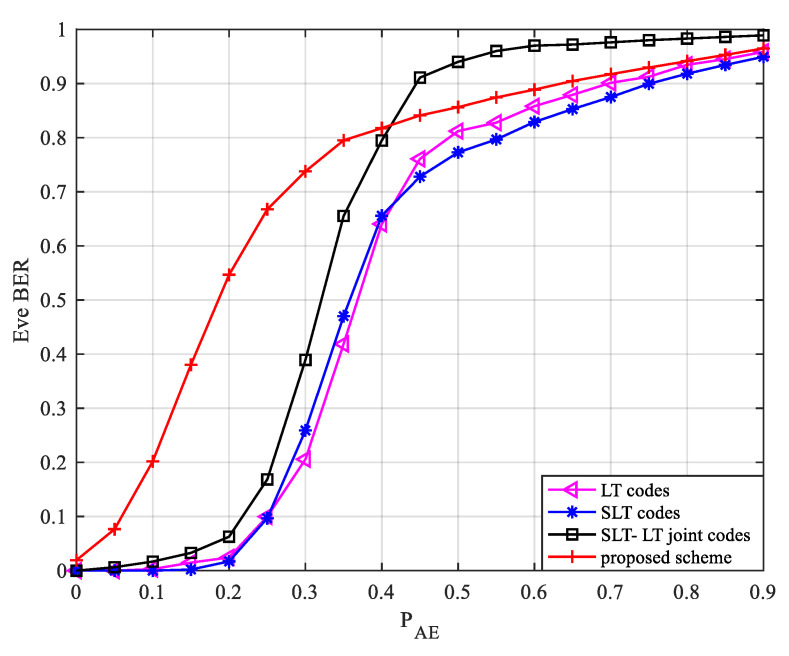
Relationship between Eve BER and erasure probability of the eavesdropping channel.

When the legitimate channel erasure probability $P_{AB}=0.3$ remains unchanged, and the eavesdropping channel environment changes, the eavesdropper’s BER is shown in Figure 6.

Figure 6 shows the relationship between the eavesdropper’s BER and the eavesdropping channel erasure probability. It can be seen from Figure 6 that when the legal channel is not dominant or has a small advantage, the scheme described in this paper can achieve a great improvement in security performance, and the closer the quality of the legal channel and the eavesdropping channel is, the more obvious the improvement of system security performance. When $P_{AB}$ remains unchanged, as $P_{AE}$ increases, the Eve BER of the four schemes all increase. When $P_{AE}=0$, the eavesdropping channel has no noise interference, Eve can receive more message symbols than Bob, and Eve also successfully decodes when Bob completes the decoding. When $P_{AE}\leq P_{AB}$ and $P_{AE}\to P_{AB}$, the BER of Eve increases rapidly in this scheme. This is because the transfer information received by Eve in this scheme is a degraded version of Bob, so even if Eve receives more codewords later, they are difficult to decode. When $P_{AE}>P_{AB}$, the eavesdropping channel is worse than the legal channel. With the increase in the erasure probability of the eavesdropping channel, the random sequences and codewords that can be intercepted by Eve become fewer and fewer, so the BER of Eve increases continuously. When $P_{AE}\to 1$, it is difficult for Eve to intercept the useful codewords for decoding. In the four schemes, the BER of Eve gradually approaches 1, and complete confidentiality can be achieved.

**Figure 7:**
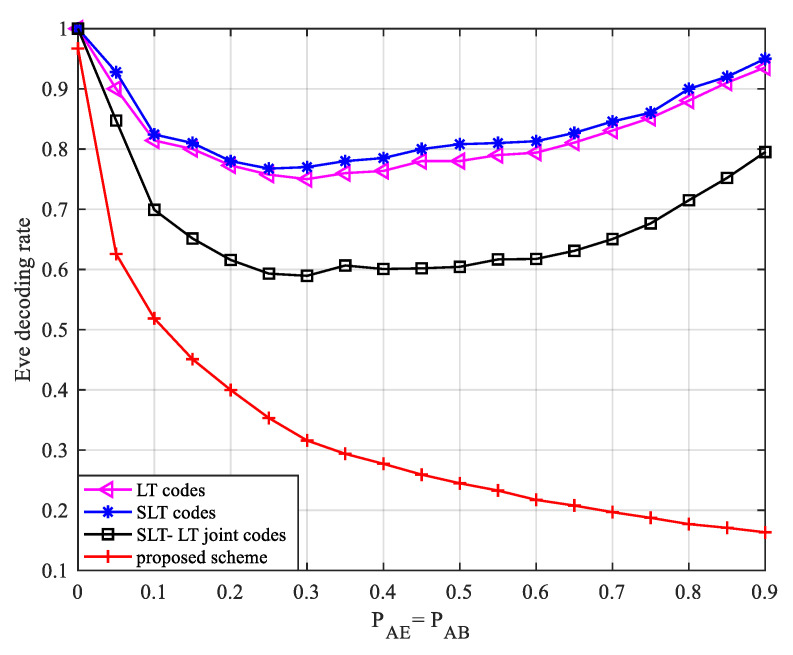
Variation in Eve’s bit error rate when the legitimate channel and the eavesdropping channel are the same.

Further, we study the change in the bit error rate of the eavesdropper when the legitimate channel and the eavesdropping channel have the same channel quality. Let $P_{AB}=P_{AE}$; the BER variation for Eve is shown in Figure 7.

It can be seen from Figure 7 that under the same channel quality, our scheme has the lowest recovery rate for eavesdroppers, followed by the SLT-LT concatenated coding scheme in Ref. [22], and the SLT scheme has the worst security performance. For the scheme proposed in this paper, since legal channel noise is introduced into the Eve end in the partial information transfer stage, the partial information symbols received by Eve are the degraded version of Bob. As the channel quality deteriorates, partial information symbols intercepted by Eve decrease rapidly, while the average degree of subsequent codewords increases, making it increasingly more difficult for Eve to decode the received codewords, so the recovery rate gradually decreases. In the other three schemes, as the probability of channel erasure increases, the difference between the codewords received by Bob and Eve gradually increases. When Bob completes the decoding, the probability that the eavesdropper has not successfully decoded continues to increase, and the decoding rate decreases; when the channel quality is very poor, both Bob and Eve need to receive more coded symbols to recover the source symbols. Eve’s decoding rate increases accordingly, and the decoding rate curves show a trend of first decreasing and then increasing. It can be clearly seen that when the quality of the main channel and the eavesdropping channel are the same, the proposed scheme has better security performance.

Summarizing, the SLT scheme based on partial information encryption transfer proposed by us can effectively improve the security of the system under the condition of small decoding overhead.

## 6. Conclusions

We propose a secure transmission scheme of SLT code based on partial information encryption transfer. In the proposed scheme, the eavesdropper introduces legitimate channel interference while intercepting the transferred encrypted information, so that the eavesdropper receives the transferred information symbol as a degraded version of the legitimate user, and the channel noise and random sequences further increase the eavesdropper’s uncertainty concerning the transferred information. At the same time, when the information source performs SLT encoding, the degree distribution is adjusted by using the number of information symbols known to legal users. Even if the eavesdropper can receive more coded symbols, they are difficult to decode because only a small number of partial information symbols are intercepted in the initial stage. Compared with the existing work, the proposed scheme still has good security performance when the legal channel is not dominant.

## Data Availability

Not applicable.
